# Modelling population responses to workplace minimum dietary standards introduced as workers return after social lockdowns

**DOI:** 10.1186/s12889-022-14729-x

**Published:** 2022-12-20

**Authors:** Benjamin J. J. McCormick, Andrea Scalco, Tony Craig, Stephen Whybrow, Graham. W. Horgan, Jennie I. Macdiarmid

**Affiliations:** 1 Rowett Institute, University of Aberdeen, Ashgrove Rd. W, Aberdeen, AB25 2ZD UK; 2 The James Hutton Institute, Craigiebuckler, Aberdeen, AB15 8QH UK; 3 Biomathematics and Statistics Scotland, Ashgrove Rd. W, Aberdeen, AB25 2ZD UK

**Keywords:** Diet, Norms, Behaviour, Spill over, Agent based model

## Abstract

**Background:**

Diet norms are the shared social behaviours and beliefs about diets. In many societies, including the UK, these norms are typically linked to unhealthy diets and impede efforts to improve food choices. Social interactions that could influence one another’s food choices, were highly disrupted during the lockdowns in response to the COVID-19 pandemic. A return to workplaces and re-establishment of eating networks may present an opportunity to influence dietary norms by introducing minimum dietary standards to in workplaces, which could then spread through wider home and workplace networks.

**Methods:**

An agent-based model was constructed to simulate a society reflecting the structure of a city population (1000 households) to explore changes in personal and social diet-related norms. The model tracked individual meal choices as agents interact in home, work or school settings and recorded changes in diet quality (range 1 to 100). Scenarios were run to compare individuals’ diet quality with the introduction of minimum dietary standards with degrees of working from home.

**Results:**

The more people mixed at work the greater the impact of minimum standards on improving diet norms. Socially isolated households remained unaffected by minimum standards, whereas household members exposed directly, in workplaces or schools, or indirectly, influenced by others in the household, had a large and linear increase in diet quality in relation to minimum standards (0.48 [95% CI 0.34, 0.62] per unit increase in minimum standards). Since individuals regressed to the new population mean, a small proportion of diets decreased toward lower population norms. The degree of return to work influenced the rate and magnitude of change cross the population (-2.4 points [-2.40, -2.34] in mean diet quality per 20% of workers isolating).

**Conclusions:**

These model results illustrate the qualitative impact social connectivity could have on changing diets through interventions. Norms can be changed more in a more connected population, and social interactions spread norms between contexts and amplified the influence of, for example, workplace minimum standards beyond those directly exposed. However, implementation of minimum standards in a single type of setting would not reach the whole population and in some cases may decrease diet quality. Any non-zero standard could yield improvements beyond the immediate adult workforce and this could spill between social contexts, but would be contingent on population connectivity.

**Supplementary Information:**

The online version contains supplementary material available at 10.1186/s12889-022-14729-x.

## Background

Diets in high income settings are typically unhealthy, with high consumption of foods high in sugar, salt and fat, [1–4]. Over several decades, considerable effort has been made seeking to improve individuals’ diets with limited success in nudging consumers to develop healthier choices [5]. Dietary norms, defined as the shared social behaviours and beliefs about diet health that form expected and acceptable behaviour [6, 7], can have a great influence on food choices as people tend to comply with the norms, which for many is an unhealthy diet. Understanding social interaction and the effects of networks is important for effective action to change diets. The workplace has been the setting for many dietary intervention studies to change food choices to improve diets with mixed success [8, 9], but there are few studies of social interactions and effects of networks within and beyond the workplace, which may exert an important influence on food choices.

In the UK, as elsewhere, many workplaces were closed between March 2020 and the summer of 2021 when lockdowns were instigated in response to the COVID-19 pandemic [10]. This dramatically altered the patterns of social interactions and therefore the extent to which people could influence one another’s food behaviours [11]. Currently, as workers gradually return to workplaces after COVID-19 isolation there is an opportunity to explore the potential consequences of setting minimum dietary standards for food served in workplace settings where adults interact and can develop shared food norms. To study this at scale could take a long time to observe an effect, would be costly and unlikely to reveal the role of social interactions in food environments. Using a simulation model is an alternative, providing qualitative insights into the consequences of complex interactions between people and how these may influence food choices, as well as insights into a large-scale intervention and behaviours in a dynamic system.

An example of a policy to coerce dietary choice and shift norms is the introduction of minimum dietary standards for the nutrient content of school meals. These have been variously introduced in different countries, but there has been little programmatic evaluation or whether school meals have changed diets overall [12]. Although these policies typically restrict meal choices to healthier options, a tangential benefit is that the school population grows accustomed to healthier attitudes to food, although it is unclear that food norms formed at school between students [13] are carried into other eating settings [14, 15]. Whilst adult diets are often more complicated given the range of eating occasions and circumstances, many workplaces have some catering provision that could regulate dietary standards of meals in a similar manner to schools.

Understanding dynamic dietary behaviours is challenging because of the non-linear interactions and potential feedback between individuals who influence one another over time. These analytical challenges are ideally met through an agent-based model (ABM) that can simulate complex systems and how individuals respond to rule-based decisions [16] based on personal, heterogeneous attributes and their interactions with one another. Here we present an ABM that simulates how meal choices are made based on reconciling personal food norms and the perception of group norms in different social settings (households and workplaces or schools). We hypothesize that (1) coercing diet standards in workplaces (adults) or schools (children) will improve diet choices through changing diet personal and group norms; (2) restricting social interactions because of working from home will reduce the impact of minimum dietary standards across the whole population; and (3) there will be ‘spillover’ of emerging diet norms as individuals transmit their adapting diet norms between their social networks [17].

## Methods

An individual-based ABM was constructed to capture the dynamic interactions between individual adults and children as they eat in different settings, home, work or school. An ABM uses rules to simulate decision-making processes as individuals influence one another through perceived norm-based dietary choices at shared eating occasions. The ABM simulates heterogeneous individuals, therefore we are able to describe how patterns of behaviour evolve from simple rules of interaction that go beyond population averages [18]. The aim is twofold: (1) to qualitatively examine the population responses across different social settings to the introduction of minimum dietary standards for meals and (2) to examine the consequences of workers gradually returning to the workplace (so called ‘hybrid’ or ‘blended’ working) following a period of strict work-from-home regulations.

### Model population

Households were generated within the model and populated with adults with a given employment status and a number of children (Table 1). The household population defined one social network and eating group for an agent (i.e. person) and a second social network was described by workplace (adults) or school (children). Workplaces were created in proportion to the number of households and stratified by their number of employees (big, medium or small; Table 1), which defined the total population size in the workplace. In order that the model is situated with plausible socio-demographic structure, the data were based on a real city, in this case Aberdeen, Scotland, but this model could be applied to any location.

**Table 1:** Model parameters and their initial values

Parameter	Value	Reference
Household Structure		
Working adult and children (%)	30.5	Office of National Statistics, https://www.nomisweb.co.uk/ (2019)
Working adult but not children (%)	54.4	
No working adults, but children (%)	2.6	
No working adults or children (%)	12.4	
Workplace size: number of employees (n workplaces)		
Big	232 (2)	Scottish Government, Business in Scotland (2017) created in proportion to the population
Medium	132 (5)	
Small	24 (7)	
Schools: number of students (n schools)		
Secondary	230 (1)	Derived from Aberdeen City Census (2011)
Primary	89 (3)	
Diet behaviour		
Mean diet quality	calculated	
Variability in diet quality	Normal (mean = 13, sd = 2.5)	Calculated from Kantar World Panel sample of 1198 Scottish households
Weight toward group matching	Triangular (min = 0, peak = 0.39, max = 1)	Based on Vartanian et al. (2015)
Empirical diets		
Diet Quality Index (median, IQR)	38.1, 26.3 to 51.3	Calculated for *n* = 837 Scottish adults from NDNS years 1–4

The model used meal-based time steps (i.e. breakfast, lunch, dinner) with three time-steps per day. For breakfasts, dinners and all weekend meals, agents were all assumed to eat within their household group. For workplace weekday lunches, workers ate in a group that was uniformly randomly sampled from co-workers at the same workplace and children similarly ate in randomised networks within their school. At each day within the model, each workplace/school had a random daily menu of food options, uniformly sampled from those that met the minimum dietary standards. Agents therefore ate with their home network at the start of each day, would take those preferences towards their food choices into the workplaces (as their personal norm), update their preference through interaction with their workplace/school social network and return home with the updated personal norm to again interact with their home social network. In households that had adults in different workplaces and children at school, this would mean multiple sources of influence were brought home for the evening meal choice and in workplaces, heterogeneous households would mix.

### Model process

**Fig. 1:**
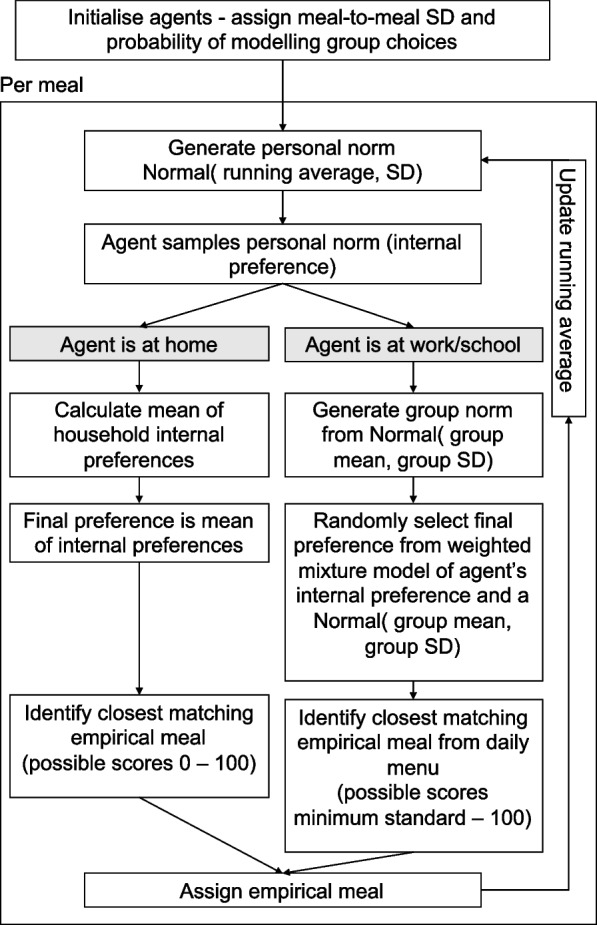
Diagram of steps for diet selection by agents. Following initialisation during which agents are assigned constant values for their meal-to-meal variability and the weight for modelling group choices, each meal agents each make an unconstrained internal preference from a random normal distribution centred on their previous choice. In the household network (breakfast and dinner) the family choice was the mean of the agents’ internal preferences. For lunches, the personal norm was combined with the group norm. The meal chosen by each agent was the item from the daily menu that most closely matched the final preference

The process of how agents select diets is illustrated in Fig. 1. In summary, agents follow a series of rules to select meals:

- Agents express an internal preference, unlimited by availability or social interactions, but based on recent experience;
- If at home, all household members average their internal preferences

or

2b. If at work, agents reconcile their personal preference with the preferences of others in their eating group
3. Agents select an empirically observed meal that most closely resembles their internal preference
4. Agents update their recent meal choices

At each meal, every individual had an internal preference that was unaffected by co-eaters, but determined by the personal norm. The personal norms for each agent was defined by an individuals’ diet behaviours built up over time and akin to their habitual intake. Here, a personal norm was described by a random normal distribution centred on their running mean of preceding choices (default to 45, approximately two weeks of meals) and with a standard deviation that was assigned at initialisation (see below). The internal preference was a random draw from the personal norm. For agents who ate on their own, for example they lived alone and did not work, their final meal selection was the same as their internal preference, i.e. their personal norm was unchanged.

A descriptive group norm [19], the observed shared diet behaviour of a group of individuals eating together, was defined in two ways depending on context: in the household (for breakfast and dinner or lunches at weekends) the mean of the internal preferences from each household member was calculated and was then assigned to all household members as their final preference assuming that they all eat the same meal. The shared norm was therefore the average of household members’ preferred meal choices. For workplace or school lunches, the group norm was characterised by a random normal distribution centred on the mean and standard deviation of co-eaters’ internal preferences. For each agent, a weighted mixture of the group and personal norms was estimated to simulate norm matching [7, 20] and this mixture distribution was sampled to produce a final meal preference.

The meal chosen was then the meal from the daily menu of the workplace/school, all choices of which had to meet the minimum dietary standards, or from an unconstrained list in the case of home meals, that most closely matched the final preference. An agent’s running average was updated with the chosen meal and this became the basis for the internal choice in the next (meal) time step.

### Data and model initialisation

In addition to the norm-based theory underpinning behaviours [21], empirical data were used to ground the model. Initial values are shown in Table 1.

The population structure was based on the tangible example of a medium sized UK city, Aberdeen, for which there was detailed census data (2011). For computational constraints, the model was populated with 1,000 households (approximately 1% of the number of households in Aberdeen). To illustrate food choices, we based meal selection on a diet quality index (DQI), which reflects the whole diet intake of macronutrients and food groups that is scaled zero to 100 (higher values indicative of higher diet quality) [22]. To score modelled meals, we sampled from the DQI calculated for observed whole diets reported from years 1 – 4 of the self-reported four-day diaries in the National Diet and Nutrition Survey rolling program (NDNS) [23], during which a nationally representative sample of 1000 individuals per year record a diet diary. The NDNS is cross-sectional and the DQI applies to the whole diet rather than individual meals or days and therefore does not have temporal variation per person. To describe how variable diet choices were per agent we supplemented this with analysis of longitudinal sample of 1988 Scottish households from the Kantar WorldPanel [24] to estimate the week-to-week DQI standard deviation (calculated over 100 weeks) of purchased food. The standard deviation of the per person DQI, i.e. household DQI divided by the number of occupants, was closely approximated by a normal distribution (mean 13.5, SD 2.5; Additional File 1) from which agents were assigned a random value at initialisation to define the variability of their unconstrained preference. We additionally assumed that agents differ in their degree of norm matching and we operationalised this with a random triangular distribution (from zero to one and with a peak at 0.39 following [25]) and assumed that this value remained constant. Sensitivity to changing the peak of the weighting distribution was evaluated (Additional file 2).

### Scenarios for minimum dietary standards

Models were run in which the minimum dietary standard was increased from zero to 80. The daily menu in schools and workplaces therefore ranged from the minimum standard to 100, but from zero to 100 for other meals (i.e. at home). Minimum standards were imposed either universally in all workplaces and schools or in all schools and workplaces of different sizes separately.

### Scenarios for returning to work

The model ran according to a narrative of UK COVID-19 restrictions, from pre-pandemic, through restrictions to easing with a gradual return to work where minimum dietary standards had been introduced for all meals served in workplace canteens: (i) time-steps zero to 100 (~ 33 days) of *status quo*, *i.e.* no minimum standards; (ii) time-steps 101 to 200 in which all adult agents are restricted to eat within their household group (*i.e*. enforced working-from-home), but that children ate lunch at school and were exposed to school-based minimum dietary standards (if any); (iii) time-steps 201–500 during which (a) new minimum standards were imposed in some workplaces and (b) a constant proportion of workers, uniformly sampled at random, stay at home each day (i.e. a gradual return to workplaces). In the first experiments, minimum dietary standards to mimic introducing rules about the type of food served in work or school settings were included. For illustration, a DQI of 60 was imposed which represents an increase of approximately 50% in the mean of the observed DQI from the NDNS data (mean 37.4, SD 18.7). Given that a DQI of 100 represents recommended whole diet intakes, the minimum score is a relatively modest standard to impose on individual meal and sensitivity analyses varying this value showed the same qualitative pattern but absolute differences in the population average. For each parameter combination, 20 iterations were run to describe stochastic variability and although this was a relatively low number of iterations the simulation runs were very similar (Additional File 3).

### Software

The model was written in Netlogo [26] and analysed in R [27].

## Results

Models of 1,000 households resulted in approximately 1600 adults and 600 children. Briefly, the simulated population followed the empirical data (Table 1) with slight random variations. For example, 84.6% of households had at least one working adult and 34% of households had at least one child. The workplaces setup meant that most adults were allocated to two big (median number of adult workers 220, IQR 211 to 263), five medium (median 142, IQR 102 162) and six small (median 26, IQR 10 to 36) workplaces; children were either in one secondary (median number of students 231, 218 to 244) or three primary (98, 77 to 98) schools according to their age.

### Responses to minimum dietary standards

**Fig. 2:**
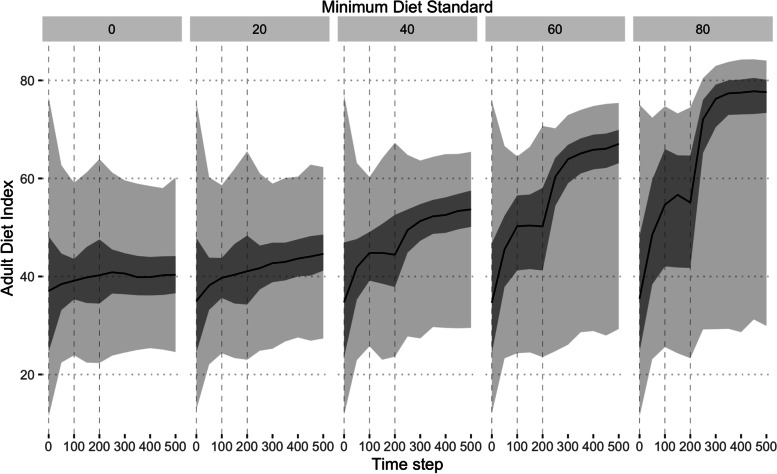
The distribution of adult diet quality over time for five different scenarios of minimum standards. Minimum standards were introduced both to schools (from time step 0) and workplaces (from time step 200). The model assumed that between workers would remain isolated between time step 100 and 200. Light grey, 2.5^th^ to 97.5^th^ percentiles; dark grey 25^th^ to 75^th^ percentiles; black line, 50^th^ percentile

The first scenario was to introduce minimum dietary standards in the form of a daily restricted menu in workplaces and schools that had to meet or exceed some threshold diet quality. Figure 2 shows the responses of the adult population to increasing the minimum standard. While minimum standards were lower than or close to 38, the median of the starting distribution for the adult diet index, there was little impact on the median of the population. However, as the minimum diet standard was increased, the median diet quality of the population also approximately linearly increased (linear regression, mean effect 0.48 [95% CI 0.34, 0.62] per 1 point increase in minimum standards, *p* < 0.001). Given that during the first 200 time steps, minimum standards were only assumed to be present in schools and then standards were introduced into workplaces as well, there were two distinct steps in the adult dietary index when adults were exposed to the new standards. The distribution the population also became increasingly skewed as the majority, but not all, of the population regressed toward the minimum standard.

**Fig. 3:**
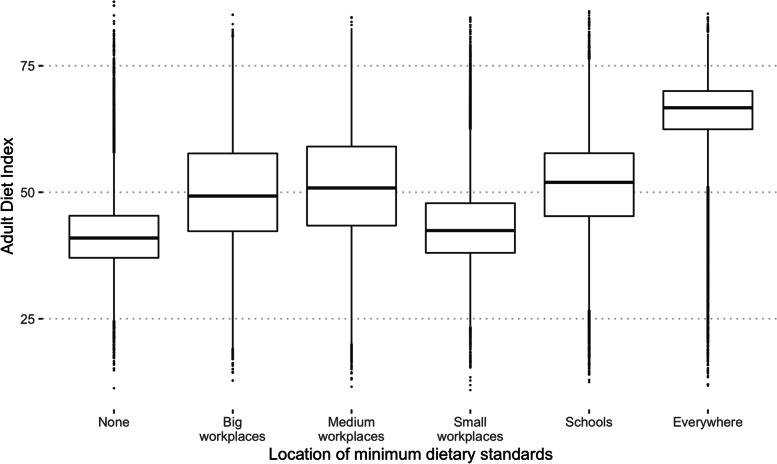
The distribution of adult diet quality in different settings. The distributions are shown after 500 time steps and assuming minimum standards of 60 in each setting

Figure 3 shows the distribution of adult dietary scores if minimum standards are introduced in different settings. The highest median scores are observed when all workplaces and schools have minimum standards. Minimum standards in big or medium sized workplaces or in schools had similar adult dietary scores, but standards in only small workplaces had little impact in any household. In this case there were fewer people to mix with and therefore be influenced by.

**Fig. 4:**
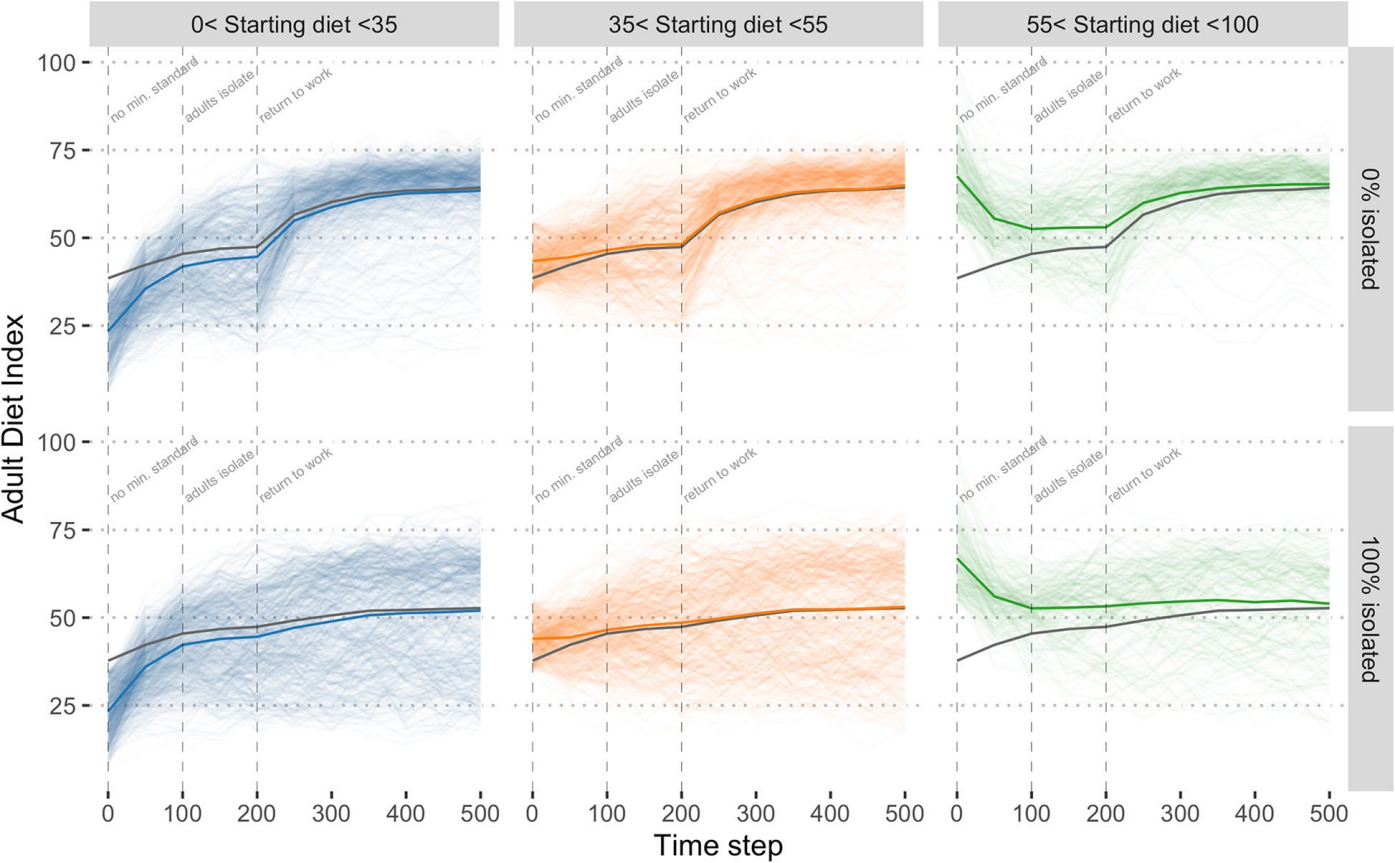
The adult diet index, stratified by starting value (columns) and isolation scenario (row). Each line indicates a single adult from one realisation of the model from a model with no minimum standards (0–100 time steps), adults isolated at home (100–200 time-steps) and followed by a return to work (top row) and minimum standards (of 60) or continued isolation at home (bottom). The population was stratified by their starting diet indices into three approximately equal groups for clarity. Thick coloured lines indicate the mean of the panel and the grey line indicates the mean of the population (*i.e.* for each row)

Figure 4 shows the results from a single iteration of the model to illustrate the trajectory of every adult from the modelled population and assuming minimum standards of 60 (in both workplaces and schools). Model iterations were highly consistent (Additional File 2) and this single iteration is representative of the patterns observed. Depending on whether individuals started below, around or above the population average, they increased, stayed approximately the same or decreased toward the population average. However, a sizeable minority of individuals who are not exposed to the intervention either through work or children at school do not converge on the population average hence the increasing breadth of the 95% confidence interval seen in Fig. 2.

### Scenarios for returning to work

**Fig. 5:**
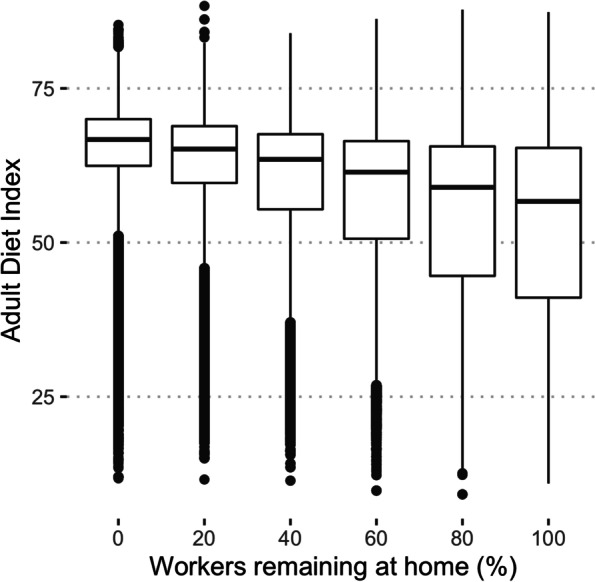
The distribution of adult diet index assuming a different proportion of workers remain in isolation. Isolated adults do not mix in the workplace to eat lunch each day. These results, shown at time step 500, assumed universal minimum standards (of 60) in both workplaces and schools

Figure 4 also contrasts two scenarios, with or without a return to work after 200 time steps. When workers return to work they mixed with one another and there was rapid convergence toward the minimum standard that resulted in a narrow (albeit skewed) distribution. If workers remain isolated (bottom row of Fig. 4) and only ate within their home group, they tend to have an approximately constant diet and did not converge on a shared norm. A secondary consequence was that when workers were isolated they were not exposed to the intervention except through children attending school and the population average was lower (mean adult diet index 63 with no isolation versus 53 with all workers isolated). The more workers who stayed at home the more attenuated the effect of the intervention (Fig. 5). There was an approximately linear trend in the mean effect of worker isolation, with a -2.36 point reduction in diet quality (95% CI -2.40, -2.34) per additional 20% workers isolated.

### Impact of social networks

**Fig. 6:**
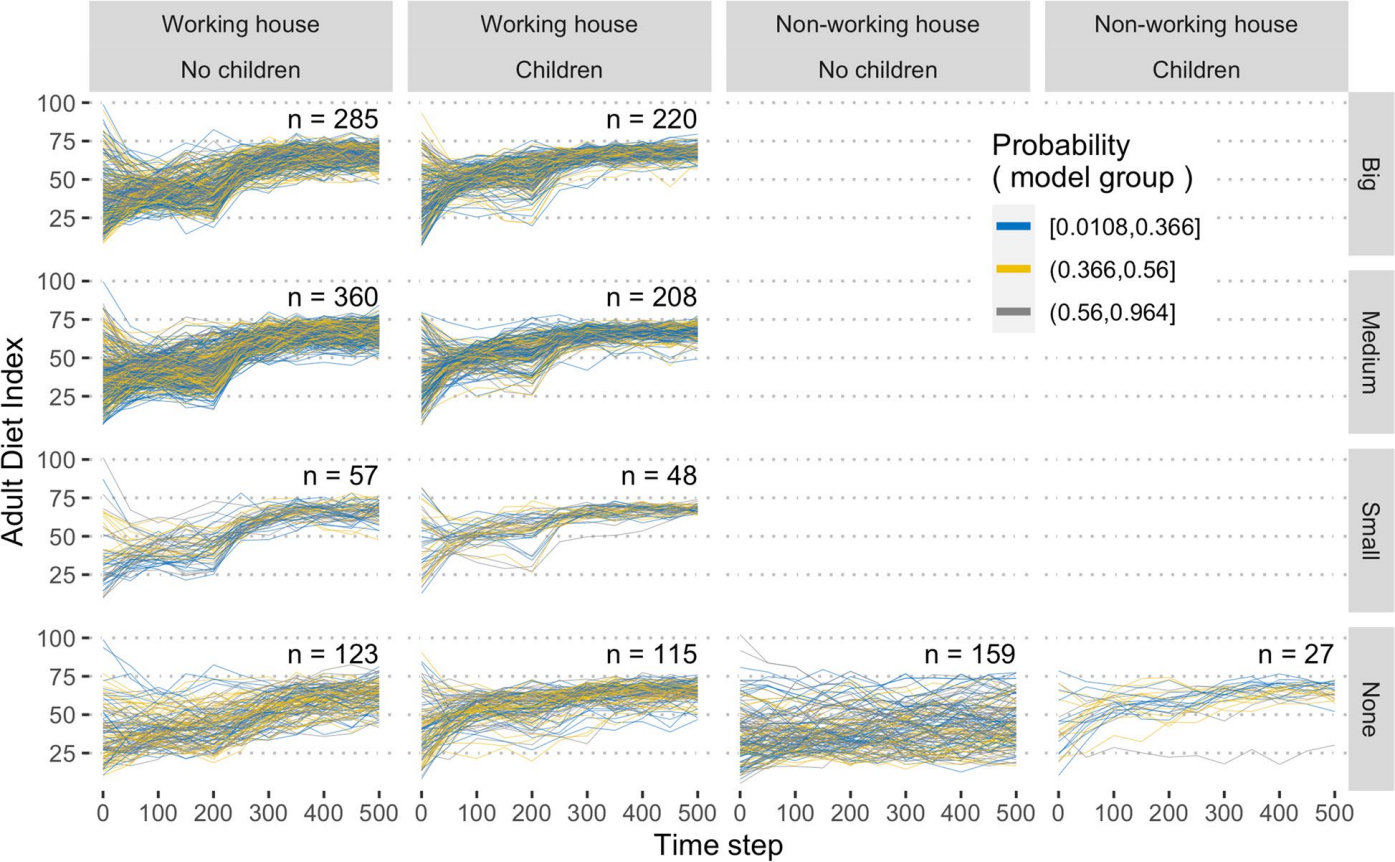
The time series of individual adult diet index stratified by household and workplace. The trajectory of every adult agent (*n* = 1602) from one realisation of the model assuming universal minimum standards of 60 is illustrated. Adult agents are stratified by whether the household contained any working or all unemployed adults and whether or not there were children present (column). They were also stratified by the size of the agent’s workplace (if any) (row) and the tertile of the weight for modelling the group norm (colour). The number of agents in each category is indicated

Figure 6 illustrates adult diet index trajectories from one iteration of the model, but stratified by the household (whether there are any working adults or children in the house) and the size of the workplace the worker in the model attends. Adults with children increased their diet standard faster than adults without children (the mean effect of the interaction between child presence and time for the first 300 days, 0.077 diet quality, 95%CI 0.059, 0.095, but thereafter the interaction was non-significant, -0.005, 95%CI -0.014, 0.003) because children are exposed to minimum standards from time step zero and they bring the updated norms back into the household, creating a positive ‘spillover’ of diet norms from school to home.

The adults from the different sized workplaces are largely indistinguishable in the distribution of diet scores or their speed of change, despite having a different number of workers in a given network (Kruskal–Wallis test, H 1.86, 2 DF, *p* = 0.4).

Assuming minimum standards in workplaces (from *t* = 200) and schools (from *t* = 0) and no working from home, adults who were exposed to minimum standards through children before those without children, achieved similar though significantly different, mean diet quality index scores at the end of the simulation (at 500 time steps mean ± SD, 67.0 ± 4.7 for adults with children in the household versus 63.1 ± 11.0 for adults without children, T test *p* < 0.01). At the end of the simulation, the difference between adult diet quality index scores for workers were significantly higher and less variable than for non-working adults (66.7 ± 5.4 for adult workers and 48.9 ± 15.4 for non-working adults, T test *p* < 0.01). However, the greatest difference was that between working adults with children in the household (67.2 ± 4.2) and adults who neither worked nor had children (46.6 ± 15.0) and it was notably more variable in addition to the difference in the mean.

Figure 6 also indicates the weight given to modelling diet choice on the group norm versus the personal norm (Pearson’s correlation 0.02). Strongly polarising the probability to either the personal or group norms had a small impact on the adult diet quality index scores (decreasing variability around the mean) but no impact on the correlation (Additional File 3).

## Discussion

A simulation of how meal choices are made by reconciling personal norms and observed group norms in household and work or school settings is presented. We hypothesized that setting minimum dietary standards in workplaces or schools would change diet norms and subsequently improve diets, and this was demonstrably the case with the magnitude of improvement in the population diet quality proportional to the minimum standards imposed. We additionally expected that if workers were isolated through, for example, working from home policies, the restricted social interactions would attenuate the impact of minimum dietary standards. This too was true and the less complete the return to work, the lower the increase in the population diet quality. Last, we hypothesised that individuals carry their changed diet norms between their social settings. The improvements in adult diets from households with children compared to those without children when minimum standards were only introduced into schools suggests that norms were both carried and transmissible between settings.

Our first scenario showed that introducing minimum dietary standards provided a positive force driving up the population norm to different magnitudes according to what was the minimum standard. Minimum standards act by truncating the distribution of meals available and removing the lowest quality options. Even a small increase in the minimum standard could have a corresponding increase in the median adult diet quality score, and our model suggested an approximately linear relationship between the two. This is noteworthy given the modest impacts observed from real-world interventions [28].

From these results, one would expect a high diet standard might yield substantial improvements in the population diet, but this could be seen as politically and practically challenging because it removes agency (individual choice) from the population [29]. The model forecasts an early rapid increase in diet quality (e.g. over the first 100 days of the model), indeed the higher the minimum standard the faster the increase. However, the since the mix of adults working from home versus returning to a work environment had a large impact on both the magnitude and speed of improving diets the precise composition of remote/home working may have implications for where and when minimum standards would be most applicable. Workplaces where people physically interact would be more amenable to change than workplaces with a greater proportion of working from home. Caution would therefore be warranted in extrapolating the impact of minimum standards given the changing patterns of how people are continuing to return to workplaces in a post-pandemic economy [30]. It is also likely that populations with a different combination workforce isolation would have different outcomes.

An advantage of the ABM is that the dynamics of the population can be tracked through time rather than, for example a pre-/post-intervention. Across all the scenarios examined, increase in diet quality saturated, reaching a plateau faster the higher the standard. This may imply that a sustained effort could be required to prevent a population from lapsing given the wide distribution of diets and tendency to regress toward the population mean. Since the current observed UK population average DQI falls significantly short of the recommended intake (DQI = 100), diet culture would need to be changed dramatically, and mechanisms to reinforce and maintain changes over time would be required.

In our results we found a phenomenon akin to social matching, copying the food choices of other eaters [20], which resulted in a strong tendency for individuals to converge toward the population mean. With a higher minimum standard, most individuals increased their diet quality over time, but some individuals decrease their diet quality toward the population average. This phenomenon has been observed and reflects social conformity [31, 32]. Offering a reference point can encourage perverse responses to attempts to shift diets [33]. But depending on the minimum standard set, the proportion of individuals who would drop in quality could be minimised. The individual-based resolution of the ABM allowed results beyond the population average to be observed, which could inform targeted approaches to future interventions and quantify the expected number of individuals that adversely respond to different minimum standards (in this case approximately half a percent per 1 point increase in standards).

Households that were more connected in the model, e.g. had more working adults in the household and children, regressed to the mean faster than households that were less connected (e.g. single unemployed adult). This illustrates a circular importance of social connectivity on shifting the population norm and the role of the population norm on changing individual dietary patterns. As an extreme illustration, households with non-working adults and no children could not be exposed to minimum standards in this model because they were not socially connected; whereas households with all adults working and children in school had the largest exposure to minimum standards and largest change in diet quality. Household structure and connectivity was important in determining the change in diet quality. When workers remained at home, they effectively became isolated from social connections and insulated from the minimum standards. In actuality, households would likely have other social interaction that might permit less direct diffusion of the changing population norm. Alternative policies to access more socially isolated households would be required to prevent ‘leakage’ of the minimum standards (e.g. through less healthy ‘meal deals’, takeaways or other sources).

Changing the probability that individuals’ conformed to the group norm rather than their personal norm had surprising little impact on the speed of convergence of the model. This may be because of a strong tendency to regress to the mean or the (very) strong group dynamic within households since we assume all household members make a single, shared meal choice. The probability of conforming to group choices is unknown, but since changing the probability distribution to extreme values had such limited impact on the population average it may not be a meaningful factor in designing policies for minimum standards given the substantial effort needed to implement it. That said, in the case of extreme group conformity, it does reduce variance in the population.

Increases in the population norm did spill over into households when minimum standards were implemented in workplaces or schools. This suggests that the direct benefits of minimum standards on the target population may be an underestimate when viewed at the population level. The indirect benefits, increases in household group norms, suggest that changing norms could be a viable policy that could shift the whole population diet quality more than targeting messages around individual choices. Given that schools are likely to be subject to policies controlling diet choices (as evidenced by many countries already imposing school-based minimum standards and emerging evidence of their impacts on child diets [34, 35]) it is notable that school-based standards in the model had a comparable effect on the adult population as workplace-based intervention. It is, however, unknown whether the long-standing minimum standards in Scottish schools (introduced in 2008, [36]) have improved adult diets, suggesting either that any spill over has been too subtle to have been detected in cross-sectional surveys to date or that spill over is an artefact in the model which is missing some factor, for instance the diversity of eating occasions and settings that also influence adult behaviours, that inhibits the spill over of norms.

This model provides a manipulatable system within which to test the consequences of population-wide and invasive policies. There are some limitations in using a modelling approach and both social connectivity and the diet environment in reality are clearly more complicated than this model assumes. This model assumes that all individuals eat meals in their respective settings, whereas adults (in particular) have access to a more complicated eating environment and many eat meals brought from home at work, eat outside the work setting (e.g. shop-bought meals, takeaways, etc.) or eat food sourced at work, but not in a group (e.g. at their desks). These results would therefore be an over-estimate the impact of social interactions even if they are qualitatively indicative of the interaction between social interactions and norms. That said, the model does not include informal interactions between households, or social interactions beyond households (for example, community or social groups and non-work friendships), which may speed up the convergence of dietary scores. Isolation of workers in the model suggests that social interactions have a large effect on changing the population norm and that acting on this could be highly beneficial. Hence, multiple interventions would be needed that would reach different groups of the population.

We based the social structure of the model on empirical data, but the model is adaptable to other circumstances. However, the diet quality of adults was similar regardless of the workplace size so the relative abundance of different sized workplaces s in different populations would not likely changes the qualitative patterns reported here. Further, in Scotland, school meals are already regulated through legislation and coordinated at a regional level so that it is unlikely that the number or size of schools would change the mixing of children and thereby the qualitative results presented here. That said, Aberdeen City has a similar distribution of schools (by size) to other Scottish cities and the proportion of the adult population that is economically active matches the national mean (Scottish census data, 2011) and so is it is likely to be a reasonable approximation to other urban centres in Scotland. The scale of the model is challenging to validate, but in time, data to empirically validate the model may emerge as the UK population emerges post-COVID and surveys of the national diet reveal short- and long-term consequences of altered eating patterns.

## Conclusions

Minimum dietary standards have the capacity to improve the population diet of those both directly exposed and indirectly exposed through social interactions. Individuals change their personal norms and thereby start to change the behaviour of others in different settings. However, as adults return to varied levels of working from home rather than in communal workplace settings, the efficacy of minimum standards could be greatly reduced. In addition, some proportion of individuals are likely to regress downward toward the mean and have decreased diet quality, though this depends on the minimum standard imposed and for how long. Given the opportunity to reform diets in a post-COVID-19 workplace, the changing landscape of how people work will likely determine how diet norms spread and consequently the effectiveness of minimum standards.

## Supplementary Information

**Additional file 1.** 

**Additional file 2.** 

**Additional file 3.** 

## Data Availability

The model code for this work is available at Zenodo, https://doi.org/10.5281/zenodo.6386946, under a Creative Commons Attribution 4.0 International licence. Code is written in Netlogo (version 6.2.1), available for Windows and OS X or Linux.

## Abbreviations

ABM: Agent Based Model
COVID-19: Coronavirus Disease
DQI: Diet Quality Index
NDNS: National Diet and Nutrition Survey
