# Supplementary material for: Modelling population responses to workplace minimum dietary standards introduced as workers return after social lockdowns
Source: BMC Public Health. 2022 Dec 20;22:2390. doi: 10.1186/s12889-022-14729-x (PMC9763797; doi:10.1186/s12889-022-14729-x)
Supplement: Supplementary file 1 — Additional file 1. [file 12889_2022_14729_MOESM1_ESM.docx]

**Modelling population responses to workplace minimum dietary standards introduced as workers return after social lockdowns**

**Additional File 1 Diet Quality Index**

*Personal variability*

Agents were each assigned a standard deviation to defined how variable their diet choices would be over time. Given unconstrained (“private”) choice, an agent’s choice was a drawn from a truncated random normal (bounded to match the 0 to 100 range of the DQI). The mean was based on the running average of previous choices, but the standard deviation was based on empirical data from the Kantar World Panel (2017-2019) (Figure S1). Household purchase data were used to estimate a per person DQI for each one of 1198 households. For each of the households, the standard deviation was calculated and generalised with a random normal distribution that was used to populate the agents in the model.

**Figure S1: The distribution of the per person standard deviation in DQI based on empirical household purchase data** (grey bars). The distribution for the model was approximated by normal distribution (black line, mean 13.5 and standard deviation 2.5).

For each meal, each agent selected the closest matching diet from the empirical National Diet and Nutrition Survey (NDNS) rolling program data based on matched age category and gender.
