# Supplementary material for: Modelling population responses to workplace minimum dietary standards introduced as workers return after social lockdowns
Source: BMC Public Health. 2022 Dec 20;22:2390. doi: 10.1186/s12889-022-14729-x (PMC9763797; doi:10.1186/s12889-022-14729-x)
Supplement: Supplementary file 2 — Additional file 2. [file 12889_2022_14729_MOESM2_ESM.docx]

**Modelling population responses to workplace minimum dietary standards introduced as workers return after social lockdowns**

**Additional File 2 Probability of matching group dietary choices**

Individual agents were assigned a constant probability of matching their meal preference to their eating group. Agents drew a random number from a triangular distribution (minimum 0, peak 0.39, maximum 1) based on matching meal sizes (Vartanian *et al*, 2015).

**Figure S3. The correlation between an agent’s probability of matching the group preference and an agent’s dietary index** was null (Pearson’s rho -0.03, -0.08 to 0.02 assuming no adult isolation; 0.03, -0.02 to 0.08 with 100% adult isolation).


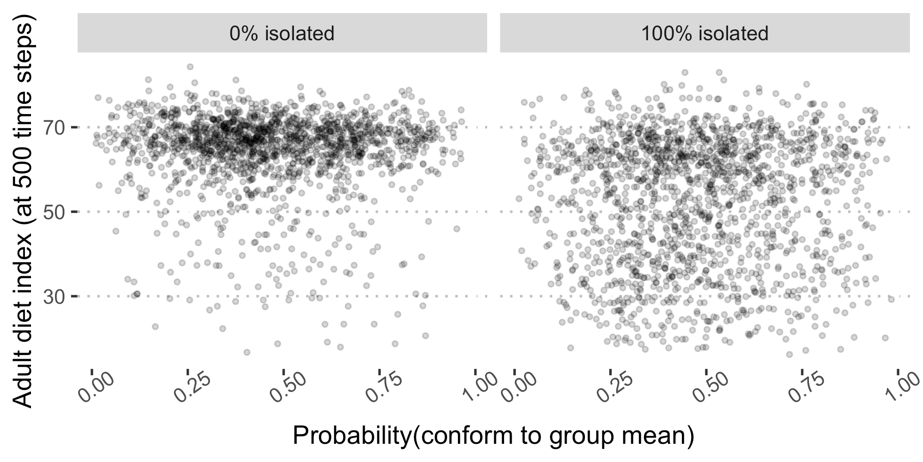


**Figure S4. In sensitivity analysis, the triangular distribution was replaced with a beta distribution that was skewed either to individuals conform to the group diet or not**.


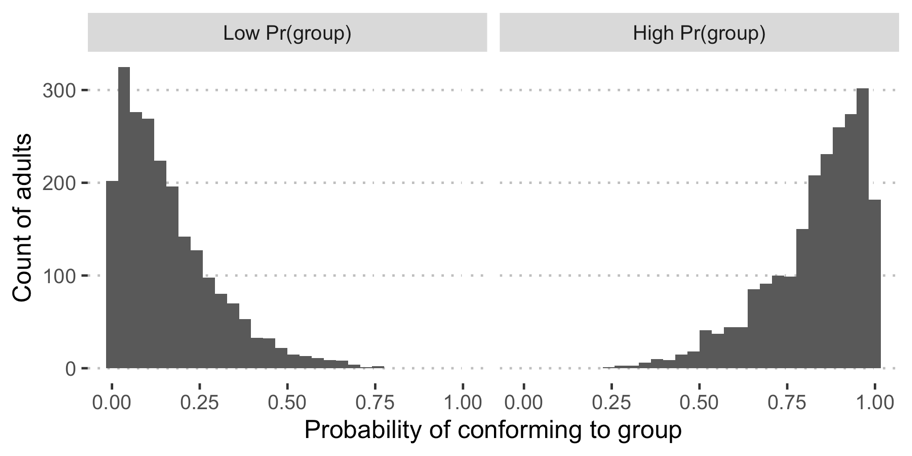


Assuming the same model structure with minimum standards of 60 in workplaces and schools, individual trajectories are illustrated below. In the low probability of group conformity (Figure S4: left; Figure S4: top row) the weight distribution was sampled from a beta distribution with shape parameters a=1, b=5; in the high conformity model (right above; bottom row below) the weights were sampled from a beta distribution with a=5, b=1. With extreme values, the difference between results is the clustering around the mean, which is tighter with greater conformity to modelling the group dietary preferences.


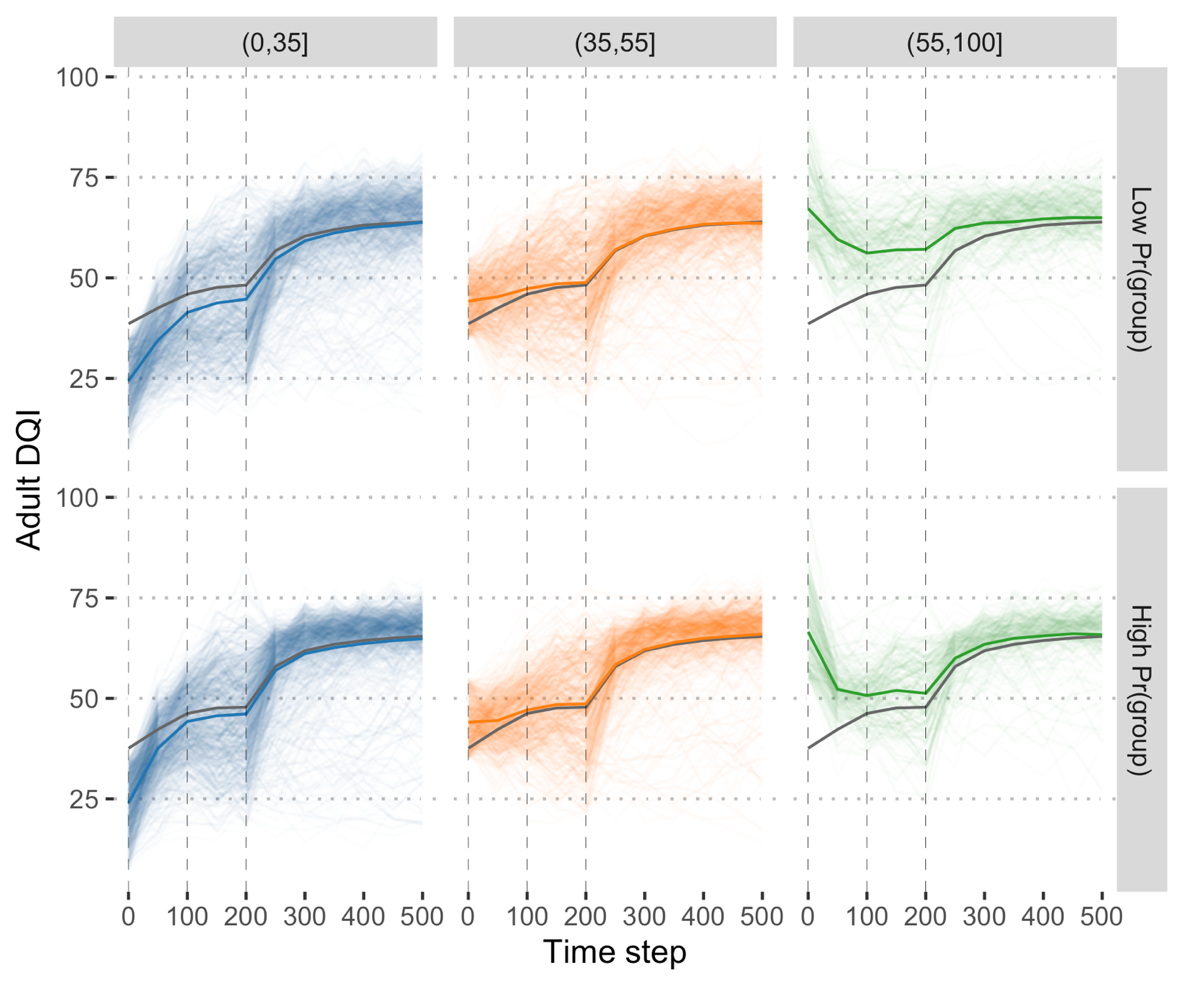


**Figure S5. Adult DQI trajectories from sensitivity analysis using a beta distribution to describe the probability that individuals conform to the group diet or not**.
