# Supplementary material for: Modelling population responses to workplace minimum dietary standards introduced as workers return after social lockdowns
Source: BMC Public Health. 2022 Dec 20;22:2390. doi: 10.1186/s12889-022-14729-x (PMC9763797; doi:10.1186/s12889-022-14729-x)

**Modelling population responses to workplace minimum dietary standards introduced as workers return after social lockdowns**

**Additional File 3 Number of iterations**

For each combination of parameters (the minimum standards and proportion of the workforce isolating) the model was repeated. The model iterations were highly consistent within a parameter set. For each parameter combination, the model was repeated 20 times to establish robust population statistics.

**Figure S2. For illustration, (A) the mean of the adult DQI was calculated after 500 time steps (the end of the model) and (B) the median and interquartile range from each of the model iterations plotted.** The difference between models was largely due to those individual agents that neither mixed through work nor through children and were therefore outliers from the majority of the distribution.

A

B


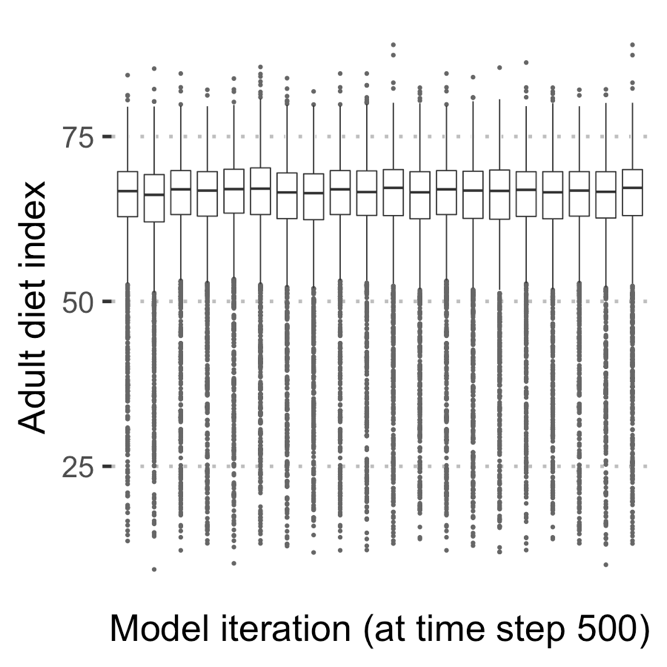

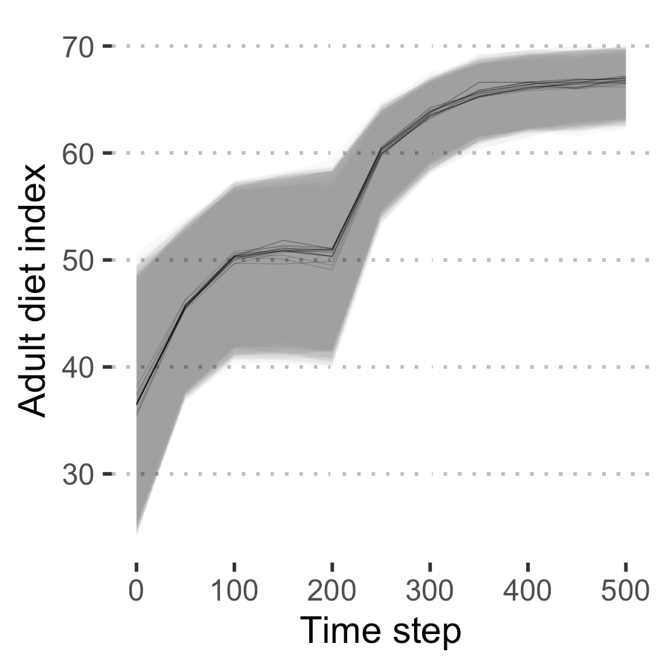

Supplement: Supplementary file 3 — Additional file 3. [file 12889_2022_14729_MOESM3_ESM.docx]
